# Rise in prescribing for anxiety in UK primary care between 2003 and 2018: a population-based cohort study using Clinical Practice Research Datalink

**DOI:** 10.3399/BJGP.2021.0561

**Published:** 2022-03-22

**Authors:** Charlotte Archer, Stephanie J MacNeill, Becky Mars, Katrina Turner, David Kessler, Nicola Wiles

**Affiliations:** Bristol Medical School, University of Bristol, Bristol.; Bristol Medical School, University of Bristol; National Institute for Health Research (NIHR) Bristol Biomedical Research Centre, Bristol.; Bristol Medical School, University of Bristol; National Institute for Health Research (NIHR) Bristol Biomedical Research Centre, Bristol.; Bristol Medical School, University of Bristol; NIHR Applied Research Collaboration West, University Hospitals Bristol NHS Foundation Trust, Bristol.; Bristol Medical School, University of Bristol; National Institute for Health Research (NIHR) Bristol Biomedical Research Centre, Bristol.; Bristol Medical School, University of Bristol; National Institute for Health Research (NIHR) Bristol Biomedical Research Centre, Bristol.

**Keywords:** anxiety, mental health, primary health care, prescribing, young adult, cohort studies

## Abstract

**Background:**

Little is known about trends in prescribing of anxiolytics (antidepressants, benzodiazepines, beta-blockers, anticonvulsants, and antipsychotics) for treatment of anxiety. Several changes may have affected prescribing in recent years, including changes in clinical guidance.

**Aim:**

To examine trends in prescribing for anxiety in UK primary care between 2003 and 2018.

**Design and setting:**

A population-based cohort study using Clinical Practice Research Datalink (CPRD) data.

**Method:**

Analysis of data from adults (*n* = 2 569 153) registered at CPRD practices between 2003 and 2018. Prevalence and incidence rates were calculated for prescriptions of any anxiolytic and also for each drug class. Treatment duration was also examined.

**Results:**

Between 2003 and 2018, prevalence of any anxiolytic prescription increased from 24.9/1000 person-years-at-risk (PYAR) to 43.6/1000 PYAR, driven by increases in those starting treatment, rather than more long-term use. Between 2003 and 2006, incidence of any anxiolytic prescription decreased from 12.8/1000 PYAR to 10.0/1000 PYAR; after which incidence rose to 13.1/1000 PYAR in 2018. Similar trends were seen for antidepressant prescriptions. Incident beta-blocker prescribing increased over the 16 years, whereas incident benzodiazepine prescriptions decreased. Long-term prescribing of benzodiazepines declined, yet 44% of prescriptions in 2017 were longer than the recommended 4 weeks. Incident prescriptions in each drug class have risen substantially in young adults in recent years.

**Conclusion:**

Recent increases in incident prescribing, especially in young adults, may reflect better detection of anxiety, increasing acceptability of medication, or an earlier unmet need. However, some prescribing is not based on robust evidence of effectiveness, may contradict guidelines, and there is limited evidence on the overall impact associated with taking antidepressants long term. As such, there may be unintended harm.

## INTRODUCTION

Anxiety disorders are common and usually managed in primary care.^1^ The National Institute for Health and Care Excellence (NICE) stepped-care guidelines recommend psychological therapy at step two, followed by the option of medication at step three.^2^ If patients progress to medication, NICE guidelines recommend antidepressant treatment. Antidepressant prescribing for any indication has substantially increased over the past two decades, which has been attributed to increasing long-term use, rather than increases in those starting medication.^3^^,^^4^ Antidepressant prescribing for generalised anxiety disorder (GAD) has also increased over the same period; however, it is not known if long-term use has similarly increased.^5^

Other drugs are also prescribed for anxiety. As a result of their potential for dependency, benzodiazepines are not recommended for long-term use.^6^ Since 2008, the number of patients prescribed a benzodiazepine in the year after a GAD diagnosis has declined.^5^ However, to the authors’ knowledge, no data have been published on prescribing for anxiety disorders in general over time. Furthermore, the 2011 NICE guidelines recommended that antipsychotics should not be prescribed for treatment of GAD.^2^ Yet, to the authors’ knowledge, there are no data on the prescribing of antipsychotics for anxiety (with the exception of GAD),^5^ or on other drugs used for anxiety — beta-blockers and anticonvulsants — in recent years.

This study examines trends in prescribing for anxiety in UK primary care between 2003 and 2018 using Clinical Practice Research Datalink (CPRD) data. The specific objectives were to:

- examine trends in prevalence and incidence of prescriptions — overall and by drug class — between 2003 and 2018, and to investigate potential differences over time according to age and sex; and
- determine whether any changes in prescribing over time were the result of: a) increases in incident prescriptions; and/or b) changes in the duration of treatment.

## METHOD

### Study population

CPRD GOLD is a large database of anonymised UK primary care electronic records. The current study used data from adults aged ≥18 years who were registered at a CPRD GOLD practice between 1 January 2003 and 31 December 2018. Patient records had to be ‘acceptable’, from practices that were ‘up-to-standard’ for at least 1 year before study entry date, and contributed data for the whole 16 years. For the analysis on incident prescriptions, patients had to have been registered with CPRD GOLD for 1 year before the first recorded anxiolytic prescription to ensure high-quality assessment of incident cases.

**Table:** How this fits in

Previous studies have found substantial increases in the prescribing of antidepressants for any indication, and for depression, over the past two decades. The current study found increases in incident prescribing for anxiety in most anxiolytic drug classes, and an increase in the number of new patients starting treatment is more likely to explain the overall increase rather than increases in long-term use. Increases in prescribing were most notable in young adults, with a marked rise in benzodiazepine prescriptions for this group. Increases in incident prescribing may reflect better detection of anxiety or an earlier unmet need; however, some of this prescribing is not based on robust evidence of effectiveness, some may contradict guidelines, and there is limited evidence on the overall impact associated with taking antidepressants long term, and therefore, there may be unintended harm.

### Codes for anxiolytics

Anxiolytic prescriptions were identified using British National Formulary codes (see Supplementary Appendix S1), compiled according to the British Association for Psychopharmacology’s recommendations for treatment of anxiety disorders^7^ and NICE guidelines.^2^ The prescription had to have occurred within the 3 months before an anxiety symptom or diagnosis code date, or the 6 months afterwards (see Supplementary Appendix S2). This aligns with a similar study.^4^

### Statistical analysis

Data analysis was conducted using Stata (version 15.1). Analyses were conducted for: any anxiolytic; any antidepressant; selective serotonin reuptake inhibitors (SSRIs) and ‘other antidepressants’; benzodiazepines; beta-blockers (propranolol); antipsychotics; and anticonvulsants (pregabalin and gabapentin).

#### Trends in prevalence and incidence of anxiolytic prescriptions

Patients entered the study on the last date of either their current registration date or the 1 January 2003, and stopped contributing person–years-at-risk (PYAR) on the earliest date of either their transfer out date; date of death; 31 December 2018; or date of their (prevalent or incident) anxiolytic prescription. PYAR was calculated separately for prevalence and incidence analyses.

For each calendar year: a) the number of patients who received at least one prescription (‘prevalent case’); and b) the number of patients who started a prescription but had no prior prescriptions of that same drug class during the study period or in the 1 year before the study start date (‘incident case’) were calculated. Annual prevalence/incidence rates were calculated by dividing the number of ‘cases’ by the total (PYAR) for each calendar year, and are presented per 1000 PYAR, with 95% confidence intervals (CIs) based on the Poisson distribution. Data were stratified by age (<25, 25–34, 35–44, 45–54, 55–64, 65–74, 75–84, and ≥85 years) and sex.

Univariable Poisson regression models were used to examine associations between year, age, sex, and prevalence/incidence of the drug(s) of interest. Prevalence rate ratios (PRRs)/incidence rate ratios (IRRs) and 95% CIs are reported. Multivariable Poisson regression models that included year, age, and sex were used to examine the independent effects of such factors. Sensitivity analyses were conducted to account for any clustering by practices within the multivariable model. To formally test whether prevalence/incidence varied over time according to age and sex, the multivariable Poisson regression model was repeated including an interaction between sex and year, and age and year.

Changes in trends were examined using joinpoint regression (version 4.7.0.0).^8^ By fitting a series of joined lines, the model selects the point(s) where the rate significantly increases/decreases (joinpoints) thus identifying the years (with 95% CI) when changes in trends occurred. Annual percentage change, based on the gradient between joinpoints, was also calculated.

Sensitivity analyses considered anxiolytic medication prescribed within 1 month before the anxiety code or 1 month afterward. Additionally, sensitivity analyses excluded patients prescribed low-dose (<75 mg) amitriptyline.

#### Trends in treatment duration

For each drug class the duration of treatment was calculated for incident prescriptions by dividing the quantity of drug prescribed by the daily dose. If no dosage instructions were entered then the median of the substance-specific prescription duration was used. Similar studies have used this approach.^3^^,^^4^ A prescription occurring within ≤12 months of the previous prescription ending was considered part of the same treatment episode. Patients not prescribed medication for >12 months were considered as having ended treatment, with any further prescriptions regarded as a new treatment episode. Duration was subdivided into categories (<15, 15–30, 31–60, 61–180, 181–365, and ≥366 days).

## RESULTS

### Sample characteristics

The dataset included 176 practices with 2 569 153 eligible patients registered across 2003–2018, and 17.7 million PYAR. There were 546 154 anxiolytic prescribing events, of which 194 049 were incident prescriptions (Table 1).

**Table 1.** Number of prescribing events during the study for any anxiolytic and by drug class

**Drug(s) of interest**	**Prescriptions, *n***	
	**Total prescribing events**	**Total incident prescribing events**
Any anxiolytic	546 154^a^	194 049^a^
All antidepressants	449 499	163 273
SSRI and ‘other antidepressants’	407 229	153 674
Benzodiazepines	210 743	94 927
Beta-blockers	100 146	52 421
Atypical antipsychotics	26 587	10 358
Anticonvulsants	28 601	14 572

a *This figure only includes one anxiolytic per year, per patient. Hence, it is not a sum of total prescribing events from each drug class. SSRI = selective serotonin reuptake inhibitor.*

### Prevalence of anxiolytic prescriptions

**Figure 1.**
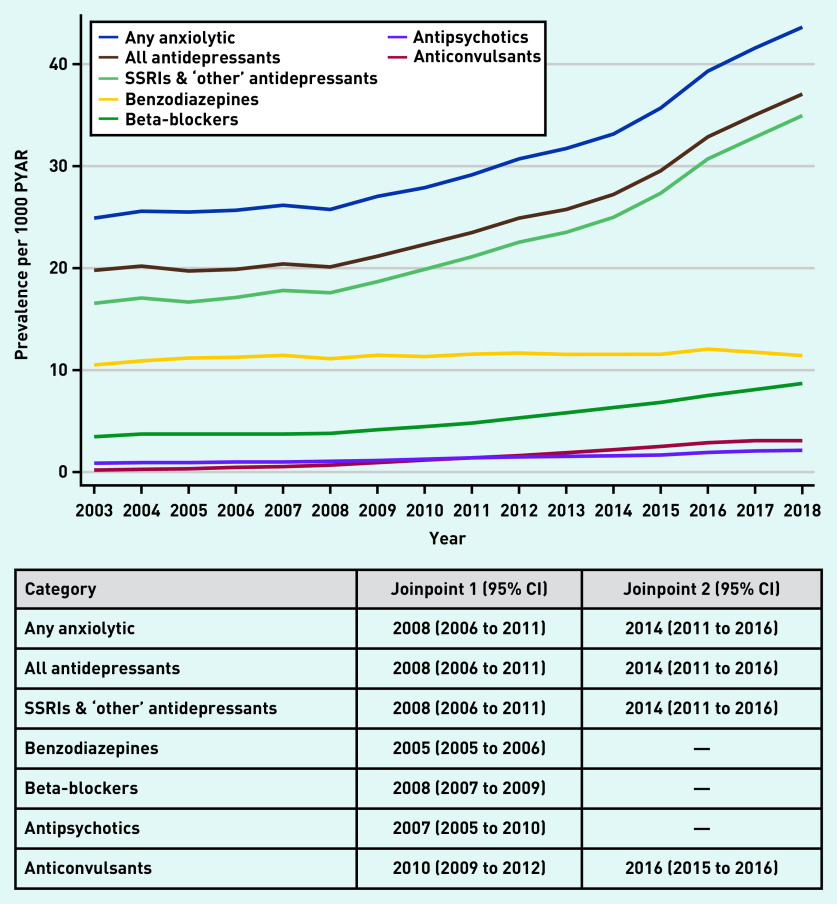
*Prevalence of anxiolytic prescriptions (any anxiolytic and by drug class) per 1000 PYAR between 2003 and 2018, with joinpoints (with 95% CI). PYAR = person–years-at-risk. SSRI = selective serotonin reuptake inhibitor.*

Estimates of the prevalence of anxiolytic prescriptions are presented in Figure 1 (and Supplementary Tables S1–S3). Between 2003 and 2008, the prevalence of any anxiolytic prescription was steady at 25–26/1000 PYAR, rising sharply to 43.6/1000 PYAR in 2018 (Figure 1 and Supplementary Table S1). Similar patterns were seen for all antidepressants, and SSRI and ‘other’ antidepressants. Prevalence of prescriptions for benzodiazepines was lower but remained steady over the 16 years, whereas beta-blockers gradually increased from 3.8/1000 PYAR in 2008 to 8.7/1000 PYAR in 2018 (Figure 1 and Supplementary Table S2). Antipsychotics and anticonvulsants were prescribed infrequently (Figure 1 and Supplementary Tables S2 and S3).

The best-fitting joinpoint models for any anxiolytic, all antidepressants, and SSRIs and ‘other’ antidepressants, included two joinpoints — one in 2008 (95% CI = 2006 to 2011), after which prevalence of prescribing increased, and one in 2014 (95% CI = 2011 to 2016), with substantial increases to 2018 (Figure 1 and Supplementary Figure S1). The best-fitting models included one joinpoint for prescriptions of benzodiazepines (2005, 95% CI = 2005 to 2006), beta-blockers (2008, 95% CI = 2007 to 2009) and antipsychotics (2007, 95% CI = 2005 to 2010), whereas anticonvulsants included two joinpoints in 2010 (95% CI = 2009 to 2012) and 2016 (95% CI = 2015 to 2016) (Figure 1 and Supplementary Figures S2–S5).

Prescribing of anxiolytics in females was over twice that of males, excluding antipsychotics (∼50% higher in females compared with males, PRR 1.46, 95% CI = 1.42 to 1.49) (see Supplementary Table S4). Prescribing of any anxiolytic, all antidepressants, beta-blockers, and SSRIs and ‘other’ antidepressants was less prevalent in older adults. In contrast, the prevalence of benzodiazepine and anticonvulsant prescriptions in those aged ≥25 years was two to three times that of those aged <25 years. The prevalence of antipsychotic prescribing for those aged 25–54 years was ∼40% higher than those aged <25 years.

The overall pattern of trends over time (any anxiolytic and most drug classes) were similar for males and females (data not shown). There was no evidence of an interaction between year and sex for antipsychotics (*P*-value for interaction 0.44), and weak evidence for anticonvulsants (*P* = 0.07). There was evidence of an interaction between year and sex for any anxiolytic (*P* = 0.02); all antidepressants (*P* = 0.007); SSRIs and ‘other’ antidepressants (*P* = 0.006); benzodiazepines (*P* = 0.03); and beta-blockers (*P* = 0.009); however, these results need to be interpreted with some caution. For the years where the interaction parameters were driving the interaction effect, there was little temporal increase in prescribing for males, such that slight increases in females represented a large relative increase, and it was this relative difference that the interaction terms were estimating. Such differences were therefore unlikely to be clinically meaningful.

**Figure 2.**
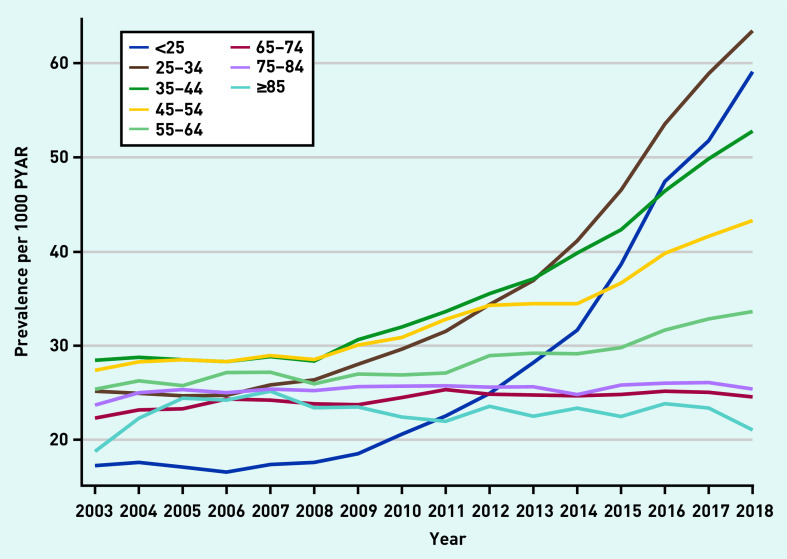
*Prevalence of any anxiolytic prescription per 1000 PYAR by age (in years). PYAR = person–years-at-risk.*

Prevalence rates were stratified by age (Figure 2 and Supplementary Figures S6–S11). Prevalence increased substantially in those aged 18–34 years in later years of the study across all drug classes. There was strong evidence of an interaction between year and age in all models (*P*-values for interaction <0.001) (Figure 2).

### Incidence of anxiolytic prescriptions

**Figure 3.**
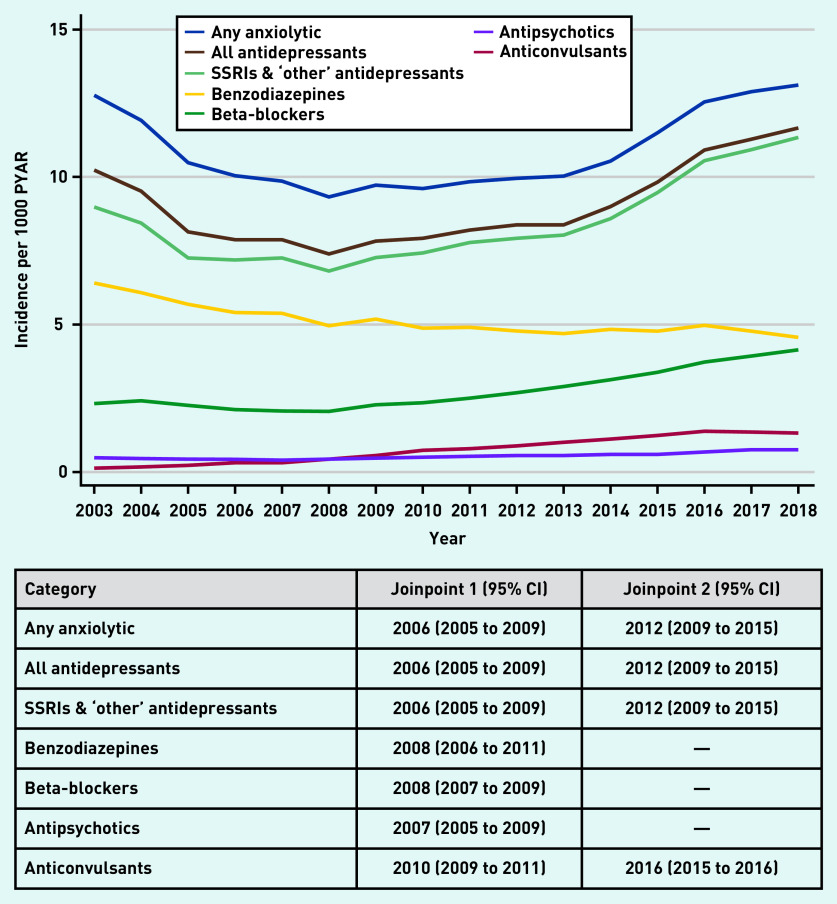
*Incidence of anxiolytic prescriptions (any anxiolytic and by drug class) per 1000 PYAR between 2003 and 2018, with joinpoints (95% CI). PYAR = person–years-at-risk. SSRI = selective serotonin reuptake inhibitor.*

The number of patients starting anxiolytics are shown in Figure 3 (and Supplementary Tables S5–S7). Between 2003 and 2006, incident prescriptions for any anxiolytic decreased from 12.8/1000 PYAR to 10.0/1000 PYAR, after which incidence remained steady until 2012, before rising to 13.1/1000 PYAR in 2018 (Figure 3 and Supplementary Table S5). Similar trends were seen for all antidepressants and SSRI and ‘other’ antidepressants. For benzodiazepines, incident prescribing declined from 6.4/1000 PYAR to 4.6/1000 PYAR between 2003 and 2018 (Figure 3 and Supplementary Table S6). In contrast, incident prescribing of beta-blockers rose from 2.3/1000 PYAR to 4.1/1000 PYAR between 2003 and 2018. The incidence of antipsychotic prescriptions was between 0.5/1000 PYAR and 0.7/1000 PYAR across the 16 years, whereas for anticonvulsants it slightly increased from 0.1/1000 PYAR to 1.3/1000 PYAR (Figure 3 and Supplementary Table S7).

The best-fitting joinpoint model for any anxiolytic included two joinpoints at 2006 (95% CI = 2005 to 2009) and 2012 (95% CI = 2009 to 2015), with similar patterns seen for all antidepressants and SSRIs and ‘other’ antidepressants (Figure 3 and Supplementary Figure S12). For benzodiazepine prescriptions, the best-fitting model had a single joinpoint in 2008 (95% CI = 2006 to 2011) (see Supplementary Figure S13). The best-fitting model had one joinpoint for beta-blockers (2008, 95% CI = 2007 and 2009) and for antipsychotics (2007, 95% CI = 2005 to 2009), and included two joinpoints for anticonvulsants (2010, 95% CI = 2009 to 2011; 2016, 95% CI = 2015 to 2016) (Figure 3 and Supplementary Figures S14–S16).

Incident anxiolytic prescriptions in females were twice that of males, excluding antipsychotics (44% higher in females compared with males, adjusted IRR 1.44, 95% CI = 1.39 to 1.50) (see Supplementary Table S8). Incident prescriptions of any anxiolytic, all antidepressants, SSRIs and ‘other’ antidepressants, and beta-blockers decreased with age. Incident antipsychotic prescriptions were slightly lower in older individuals compared with younger individuals. In contrast, those aged ≥25 years had between a 16% and 48% increased rate of incident benzodiazepine prescriptions compared with those aged <25 years. Incident anticonvulsant prescriptions in those aged ≥25 years were two to three times that of those aged <25 years.

The overall pattern of trends over time (any anxiolytic and most drug classes) were similar for males and females. There was no evidence of an interaction between year and sex for beta-blockers (*P*-value for interaction 0.40) and antipsychotics (*P* = 0.53), and weak evidence for anticonvulsants (*P* = 0.11). There was evidence of an interaction between year and sex for any anxiolytic (*P*<0.001); all antidepressants (*P*<0.001); SSRIs and ‘other’ antidepressants (*P*<0.001); and benzodiazepines (*P*<0.001), however, as previously discussed for prevalent prescribing, differences were small and unlikely to be clinically meaningful (data not shown).

**Figure 4.**
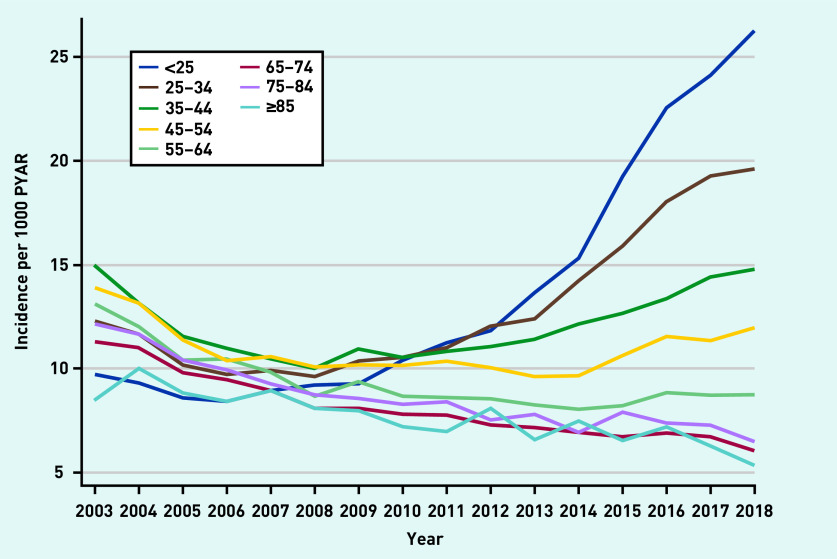
*Incidence of prescriptions of any anxiolytic per 1000 PYAR by age (in years). PYAR = person–years-at-risk.*

Incidence rates were stratified by age (Figure 4 and Supplementary Figures S17–S22). Incidence increased in those aged 18–34 years in later years of the study (2013/2014–2018) across all drug classes. There was strong evidence of an interaction between year and age for all models (*P*-value for interaction <0.001) (Figure 4).

### Sensitivity analyses

Sensitivity analyses examined the potential impact of clustering within GP practices and the impact on findings when prescriptions were restricted to 1 month either side of an anxiety code, or when low-dose amitriptyline was excluded. Trends were comparable with the main analysis.

### Trends in treatment duration for patients starting anxiolytics by drug class

**Figure 5.**
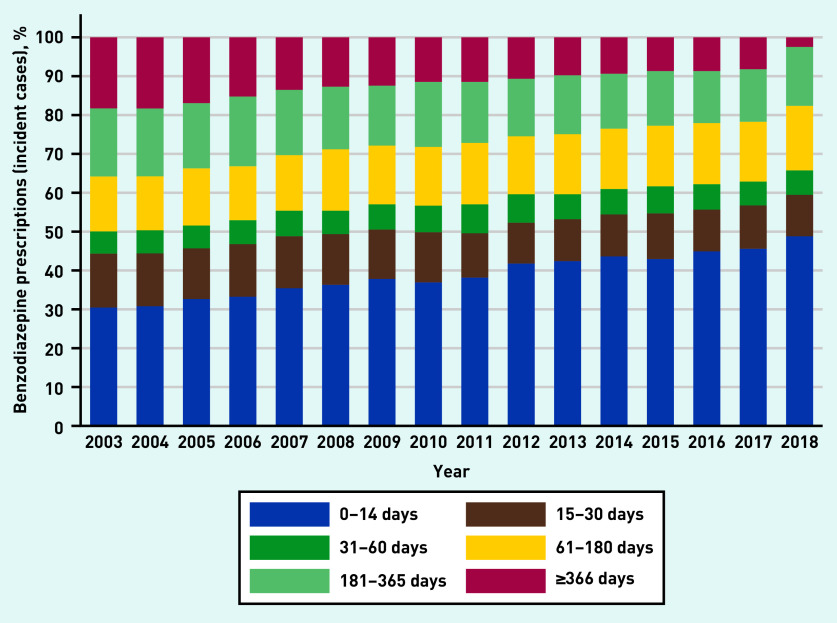
*Changes in the proportion of patients with different treatment lengths for benzodiazepines between 2003 and 2018. Data were extracted in July 2019, therefore it is likely that the figures for 2018 for the longer-duration categories are an underestimate and should be interpreted with caution.*

For all antidepressants, SSRIs and ‘other’ antidepressants, and beta-blockers, prescription duration remained relatively stable between 2003 and 2018, with year-to-year fluctuations in the duration of antipsychotic and anticonvulsant prescriptions (see Supplementary Figures S23–S27). In contrast, the proportion of short-term benzodiazepine prescriptions increased with time, whereas long-term use decreased (Figure 5). However, 44% of the prescriptions in 2017 were longer than the recommended maximum (4 weeks).^2^

## DISCUSSION

### Summary

Prevalence of prescriptions of any anxiolytic and all drug classes increased over the study period, with a marked increased from 2008 to 2018, excluding benzodiazepines, which remained steady. Incidence of prescriptions of any anxiolytic, driven by antidepressant prescribing, decreased between 2003 and 2006, after which rates remained steady, before increasing substantially between 2012 and 2018. In contrast, incidence of prescriptions for benzodiazepines gradually declined over the 16 years. Although prescribed infrequently, incidence of prescriptions of beta-blockers, antipsychotics, and anticonvulsants gradually increased from 2003 to 2018. The increases in incident prescriptions are more likely to explain the increases in prevalence, rather than longer treatment duration.

Prevalence and incidence of prescriptions in females were nearly twice that of males for any anxiolytic and each drug class, except for antipsychotics. Trends over time were similar for males and females. Prevalence and incidence of prescriptions of any anxiolytic and each drug class increased substantially in those aged 18–34 years between 2013/2014 and 2018, with a marked rise in incident benzodiazepine prescribing for this group (see Supplementary Figure S8 and S19), despite an overall reduction in incident benzodiazepine prescribing.

### Strengths and limitations

The use of a large, nationally representative dataset enabled analysis of trends over a 16-year period in terms of the prevalence/incidence of prescriptions by drug class, and by age and sex. An extensive list of anxiolytic medication was used and the prescription had to have occurred within the 3 months before an anxiety code or the 6 months afterwards.

The study was restricted to patients who had a recorded anxiety code and anxiolytic prescription. Data from patients who had been prescribed an anxiolytic but did not have an anxiety code were not captured. Furthermore, although prescriptions must have occurred within the defined time period of an anxiety code, some of these drugs may have been prescribed for other indications. Consequently, the reported figures may be an overestimate. No information was available on dispensing, adherence, or other treatment access.

### Comparisons with existing literature

Previous research has found substantial increases in prescribing of antidepressants — for any indication and for depression^3^^,^^4^^,^^9^ — but this was attributed to increasing long-term use of antidepressants rather than increasing incident prescribing. In contrast, the present study focused on prescribing for anxiety and found increases in incident antidepressant prescribing from 2013 to 2018. This is consistent with previous research that found increases in antidepressant prescriptions for GAD (issued in the year after diagnosis) between 1998 and 2018.^5^ Qualitative research has found GPs are more likely to use diagnostic codes when anxiety is severe^10^ and are more likely to prescribe an anxiolytic when a patient has a diagnosis of anxiety.^11^ Indeed, the trends in anxiolytic prescribing over time reported in the present study are very similar to trends in diagnostic codes used by GPs over the same period.^11^ Taken together this may explain the trends observed, although it is not known whether the increase in incident prescribing reflects increased awareness of anxiety among GPs, increased awareness of anxiety among patients, or a true increase in anxiety.

Previous data have shown that the prevalence of primary care benzodiazepine prescribing — for all indications — was relatively constant between 2008 and 2012.^12^ In the present study, although prevalence of benzodiazepine prescribing for anxiety was steady over time, incident prescribing decreased, excluding those aged 18–34 years. Others have reported a similar decline in the year after a GAD diagnosis.^5^ However, although long-term benzodiazepine treatment declined over time, 44% of the prescriptions in 2017 were for longer than the recommended maximum (4 weeks).^2^

The increasing prevalence and incidence of beta-blocker prescriptions in the present study is consistent with data reporting prescribing trends for all indications.^13^ Although propranolol is licensed for anxiety^14^ there is no conclusive evidence for its effectiveness,^15^^,^^16^ and it is not recommended in NICE guidance. Previous research on anticonvulsant prescribing — for any indication — found increases in prevalence of prescriptions for anticonvulsants between 2007 and 2017.^17^ In the present study there were similar trends in the overlapping years. Although the 2011 NICE anxiety guidelines state *‘do not offer an antipsychotic for the treatment of GAD’*,^2^ antipsychotic prescribing rates increased after 2011. Although some of these prescriptions may have been for other indications, previous research found that ∼50% of primary care antipsychotic prescriptions were for non-psychotic disorders, including anxiety.^18^ In contrast to the present study, another study identified slight declines in antipsychotic prescribing (1998–2018) in the year after a GAD diagnosis.^5^ However, the study by Slee *et al* linked prescriptions to anxiety codes using a wider time interval (1 year after incident diagnosis)^5^ than the present study.

Anxiolytic use in females was twice that of males and this is consistent with other studies.^3^^,^^19^^,^^20^ Previous research found that the prescribing of antidepressants (any indication) increased with age.^3^^,^^20^ In contrast, the present study found that antidepressant prescribing for anxiety was less prevalent in older adults, with the largest increase in young adults, most notably in those aged <25 years. This may be because younger patients are more likely to present with anxiety,^21^ with GP interview data suggesting this increase may be driven by increasing use of social media and increasing pressure on young people.^11^ It is possible that the increases in prescribing for young adults may be the result of earlier unmet need. Referrals to child and adolescent mental health services (CAMHS) have increased in recent years.^22^ However, in 2018–2019, over a quarter of referrals to CAMHS were rejected, and, for those who access treatment through CAMHS, there is a lack of support during the transition from CAMHS to adult mental health services.^23^ These factors may, in part, be contributing to the substantial rise in anxiety and its pharmacological treatment among young adults over the past decade.

### Implications for research and practice

Increases in incident prescribing for anxiety, especially for young adults, may reflect better detection of anxiety, increasing severity of symptoms or earlier unmet need. However, some of this prescribing is not based on robust evidence of effectiveness, and may contradict guidelines. It is known that once people have started taking antidepressants they often continue long term, and there is increasing evidence that this may be associated with unintended harms. The rise in prescribing of antidepressants for anxiety in young adults (aged <25 years) has been substantial in recent years. Although incident benzodiazepine prescribing fell over time, increases have been seen in those aged <35 years. In 2017, 44% of benzodiazepine prescriptions were longer than the recommended maximum of 4 weeks. Research is needed to improve understanding about why this is happening and to provide interventions that are acceptable and effective for young adults that can mitigate the growing reliance on pharmacotherapy for this age group.

## Data

The CPRD dataset reported in this work is not publicly available due to a lack of informed consent and ethical approval for open access, but can be requested from CPRD. Requests require a protocol describing the purpose of data access, ethical approval at the applicant’s institution, and provision for secure data access.
